# Workplace Violence Against Nurses and Its Association With Mental Health and Turnover Intention: A National Cross‐Sectional Study

**DOI:** 10.1155/jonm/2818047

**Published:** 2026-01-17

**Authors:** Jiaqing He, Jiaxin Yang, Jianghao Yuan, Qiang Yu, Elihuruma E. Stephano, Wenjia Zhang, Yamin Li, Yusheng Tian

**Affiliations:** ^1^ Clinical Nursing Teaching and Research Section, The Second Xiangya Hospital of Central South University, Changsha, Hunan, China, csu.edu.cn; ^2^ National Clinical Research Center for Mental Disorders, The Second Xiangya Hospital of Central South University, Changsha, Hunan, China, csu.edu.cn; ^3^ Department of Psychiatry, The Second Xiangya Hospital of Central South University, Changsha, Hunan, China, csu.edu.cn; ^4^ School of Computer Science and Engineering, Central South University, Changsha, Hunan, China, csu.edu.cn; ^5^ Xiangya Nursing School, Central South University, Changsha, Hunan, China, csu.edu.cn; ^6^ Department of Neurosurgery, The Second Xiangya Hospital of Central South University, Changsha, Hunan, China, csu.edu.cn; ^7^ Institute of Education, Psychology and Human Development, University College London, London, UK, ucl.ac.uk; ^8^ Hunan Provincial People’s Hospital, The First-Affiliated Hospital of Hunan Normal University, Changsha, Hunan, China, hunnu.edu.cn

**Keywords:** cross-sectional study, mental health, nurse, turnover intention, workplace violence

## Abstract

Hospital workplace violence is a significant global public health concern, impacting nurses’ mental well‐being and their likelihood of leaving their jobs. Previous research explored the associations between workplace violence, mental health, and turnover intention among nurses, which yielded inconsistent results due to smaller sample sizes, thus highlighting the need for a more comprehensive investigation with a large sample of a representative population. This study, drawing on a large dataset of 116,345 nurses from 67 tertiary hospitals across 31 provinces in China, aimed to assess the relationship between workplace violence and nurses’ mental health and turnover intentions between October and December 2023. Data were analyzed using multilevel regression models. The study assessed the varying levels of workplace violence (low, moderate, and high), mental health outcomes, and turnover intention. The study found that a substantial number of nurses, 30,987 (26.6%), experienced workplace violence in the year prior to the survey, with varying levels of severity. Specifically, 27,225 (23.4%) encountered low‐level violence, 3519 (3%) moderate violence, and 243 (0.2%) high‐level violence. After controlling for sociodemographic and work‐related variables, workplace violence was significantly associated with depressive symptoms (*p* < 0.001), stress (*p* < 0.001), anxiety symptoms (*p* < 0.001), burnout (*p* < 0.001), and turnover intention (*p* < 0.001). These findings underscore the critical need for policymakers, hospital administrators, and supervisors to take proactive measures. It is essential to implement strategies that both reduce the incidence of workplace violence and provide robust psychological support and interventions for nurses who have been affected. This collaborative effort will be crucial in protecting the mental health of nursing professionals and fostering a safer, more supportive work environment.

**Trial Registration:** Chinese Clinical Trial Registry: ChiCTR2300072142

## 1. Introduction

Workplace violence (WPV) is defined by the US Occupational Safety and Health Administration as “an act or threat involving physical violence, harassment, intimidation, or other disruptive and threatening behavior that takes place at the workplace” [1]. The healthcare sector is disproportionately affected, accounting for 73% of all WPV incidents in the United States [2]. This is a global public health concern, with a meta‐analysis of 47 studies showing that 62.4% of healthcare workers (HCWs) in China have experienced at least one form of WPV [3].

Nurses, who constitute nearly half of the global healthcare workforce and are crucial members of interdisciplinary teams, face a significantly elevated risk of WPV, as shown by the National Institute for Occupational Safety and Health [4]. This heightened vulnerability stems from their direct and continuous interaction with patients and their families, who may become aggressive due to illness, injury, or stress [5]. Additionally, the dynamics of nurse–patient interactions have become more complex and strained, partly due to limitations in medical resources [6]. A significant challenge is the underreporting of WPV incidents by nurses, often driven by fear of retaliation, the absence of visible injuries, complex reporting processes, or a perceived lack of support from management [7]. This culture of silence and compliance can lead to profound negative emotional consequences for nurses, including feelings of fear, anger, and diminished self‐esteem [8].

WPV against HCWs creates a cascade of serious consequences that threaten both individual well‐being and healthcare system stability. The immediate physical impacts range from minor injuries such as bruises to severe trauma requiring hospitalization, and in extreme cases, death [9, 10]. Beyond physical harm, the psychological consequences, including depression symptoms [11], anxiety symptoms [12], stress [13], and suicide [14], are equally devastating and well‐documented across multiple studies. A comprehensive scoping review identified WPV as a significant risk factor for mental health problems among nurses [15], with those experiencing both physical and nonphysical violence showing higher rates of depressive and anxiety symptoms [16]. A crisis of professional identity further compounds the psychological burden, as nurses subjected to violence often experience diminished social regard and understanding, which intensifies their depression and anxiety symptoms [17].

These psychological stressors from WPV contribute to a critical occupational hazard, burnout. The persistent stress and aggressive encounters increase burnout levels, characterized by physical and mental exhaustion, workplace alienation, negative attitudes toward work, and perceived loss of professional efficacy [18]. This condition has reached alarming proportions, with a meta‐analysis of 94 studies revealing a 30% prevalence of burnout symptoms among nurses [19]. The job demand‐resource model explains this phenomenon, showing how excessive job demands, including high work pressure, interpersonal conflicts, and hostile work environments, directly contribute to burnout [20].

The ripple effects extend far beyond individual nurses to impact the quality of healthcare delivery [21, 22]. Considering these findings, it is essential to evaluate and meet the mental health needs of nurses who are subjected to WPV, ensuring they receive prompt and effective psychological support. In addition, previous research has demonstrated that deterioration in care quality, combined with reduced employee motivation and engagement, leads to increased absenteeism and higher turnover rates [23, 24]. These workforce disruptions occur against a backdrop of existing global nursing shortages. The World Health Organization projects a critical shortfall of 4.5 million nurses worldwide by 2030 [25]. Even in China, where the nursing workforce expanded from 1.22 to 4.10 million between 1998 and 2018 at an annual growth rate of 6.3%, national shortages persist due to geographic inequities and high turnover rates [26]. Alarmingly, nearly 50% of nurses report intentions to leave the profession, a rate significantly exceeding the global ICU turnover average of 27.7% [27, 28]. Without intervention, these trends threaten to intensify workforce shortages and create additional strain on healthcare systems.

Despite extensive literature exists on the general impact of WPV, critical knowledge gaps persist in understanding its specific effects on nurses’ mental health and turnover intentions [29, 30]. Moreover, existing studies suffer from significant methodological limitations, including small sample sizes and single‐center designs that severely restrict the generalizability of findings. This limited evidence base creates substantial barriers for policymakers and healthcare leaders seeking to develop comprehensive, evidence‐based interventions to address WPV among nurses. This will help to meet the United Nations Sustainable Development Goal (SDG) Target 3.8.1, which emphasizes accessing quality essential healthcare services for all by 2030 [31]. Therefore, in this study, we conducted a multicenter, large‐sample investigation of whether mental health (depressive symptoms, anxiety symptoms, perceived stress, burnout) and turnover intention among nurses were associated with an increased frequency and severity of WPV.

## 2. Materials and Methods

### 2.1. Study Design and Settings

The Nurses’ Mental Health Study (NMHS) is a prospective cohort study comprising nurses from 67 tertiary hospitals in 31 administrative areas (henceforth referred to as “provinces”), which provided baseline data for the current investigation. This study adhered to the Strengthening the Reporting of Observational Studies in Epidemiology (STROBE) requirements.

The initial survey is registered with the Chinese Clinical Trial Registry.

Ethics approval was granted by the ethics committee of the lead unit (NO: LYF20230048).

### 2.2. Participants and Data Collection

The baseline recruiting process employed the cluster sampling method. Once a hospital was chosen, all of the nurses within that hospital were invited to participate. The nurses’ eligibility requirements included: (1) being over 18 years old; (2) they had to be registered nurses with formal hospital employment; and (3) consent to take part in the NMHS study. Exclusion criteria included: (1) retired, on sick or maternity leave and (2) nursing students.

The questionnaires were developed using the “Wenjuanxing” platform (https://www.wjx.cn/). Before data collection, the primary investigator trained two researchers with master’s degrees in nursing who were considered capable of explaining the purpose of the NMHS study and the specifics of each questionnaire item. Letters were then initially addressed to the nursing department of the chosen hospitals. Following consent to participate, web links to the surveys were shared with prospective participants via WeChat. Nurses were provided with an informed consent sheet outlining the purpose of the NMHS study, their right to withdraw at any time, and assurance that participation would not negatively impact their profession or personal life before they began filling out the surveys.

Between October and December 2023, 147,832 participants were invited to participate in the NMHS study. Of these, 135,161 online surveys were returned and underwent quality assessment. Two investigators independently reviewed each survey, applying a standardized quality control criterion, any questionnaire containing low‐quality data for more than three variables was classified as low‐quality and excluded. Low‐quality data were defined as: (1) Data exhibiting evident logical errors, (2) Outliers, defined as values outside of the mean ± 3SD or values lower than P25‐3IQR and higher than P75 + 3IQR. After excluding 2251 repetitive and low‐quality questionnaires, 132,190 nurses were included in the NMHS’s baseline data, with a response rate of 89.9%.

**Figure 1:**
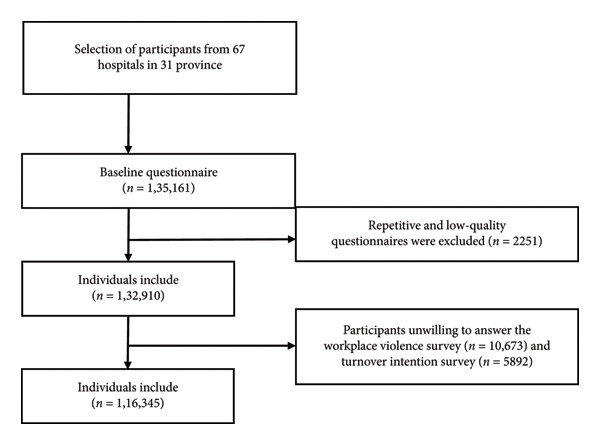
Flowchart of the NMHS study participants.

Additionally, 16,565 participants chose “prefer not to answer” in the WPV and turnover intention questionnaires and were excluded from the analysis. Finally, 116,345 participants were included in this study. The specific procedure is outlined in Figure 1.

### 2.3. Instruments

#### 2.3.1. Demographic and Work‐Related Data

Participants provided basic demographic data, including sociodemographic characteristics (sex, age, marital status, educational level) and work‐related characteristics (professional title, night shift, type of work, weekly working hours, years of experience).

#### 2.3.2. Workplace Violence Scale (WVS)

WPV was measured using the Chinese version of the WVS, which is composed of five dimensions: physical assault, emotional abuse, threat, verbal sexual harassment, and sexual assault. Each item is scored on a 4‐point scale based on how often respondents experienced WPV in the past year (0 = 0 times, 1 = 1 time, 2 = 2 or 3 times, and 3 = 4 or more times). The total scores range from 0 to 15, with higher scores indicating a more severe level of violence experienced.

The WVS has demonstrated good reliability and validity (Cronbach’s alpha = 0.92) among Chinese healthcare personnel [32]. In this study, the four levels of WPV were categorized according to the total score of the WVS (0 = none, 1 to 5 = low‐level WPV, 6 to 10 = moderate‐level WPV, and 11 to 15 = severe‐level WPV).

#### 2.3.3. Patient Health Questionnaire‐9 (PHQ‐9)

PHQ‐9 is a 9‐item self‐report questionnaire that measures the severity of depression [33]. The items are rated on a 4‐point scale: 0 = not at all, 1 = several days, 2 = more than half the days, 3 = nearly every day. Scores span from 0 to 27, with higher scores indicating more severe depression over the preceding 2 weeks. Cronbach’s alpha for the scale in the study was 0.899.

#### 2.3.4. Generalized Anxiety Disorder‐7 (GAD‐7)

The GAD‐7 is a 7‐item self‐report questionnaire that assesses the severity of generalized anxiety [34]. Participants were asked how often they experienced symptoms in the preceding 2 weeks. Each item is scored on a 4‐point scale from 0 (not at all) to 3 (nearly every day). The scores range from 0 to 21, with higher scores indicating greater symptom severity. Cronbach’s alpha for the scale in the study was 0.94.

#### 2.3.5. The Perceived Stress Scale‐4 Item Version (PSS‐4)

PSS‐4 consists of four items used to assess psychological stress over the past month [35]. The response format is a 5‐point scale ranging from 0 (never) to 4 (very often). Items 2 and 3 are reverse‐scored. The total scores range from 0 to 16, with higher scores indicating a higher perceived level of stress. Cronbach’s alpha for the scale in the study was 0.657.

#### 2.3.6. Burnout

A single question taken from the Physician Worklife Study [36] was used to assess burnout: “During the past year, have you experienced burnout?” (1 = I enjoy my work, I have no symptoms of burnout, 2 = Occasionally I am under stress, and I don’t always have as much energy as I once did, but I don’t feel burned out, 3 = I am definitely burning out and have one or more symptoms of burnout, such as physical and emotional exhaustion, 4 = The symptoms of burnout that I’m experiencing won’t go away, I think about frustration at work a lot, 5 = I feel completely burned out and often wonder if I can go on, I am at the point where I may need some changes or may need to seek some sort of help). Responses of 3, 4, or 5 were considered indicative of burnout. Responses of 1 or 2 were coded as no burnout.

#### 2.3.7. Turnover Intention

Turnover intention was assessed using a single question: “In the past year, how frequently have you had the intention to leave your job?” Participants responded on a 4‐point scale where 0 = never, 1 = rarely, 2 = occasionally, and 3 = frequently. For analysis purposes, any response indicating some level of turnover intention (scores of 1, 2, or 3) was classified as positive for turnover intention, while only those who responded “never” (score of 0) were classified as having no turnover intention.

### 2.4. Statistical Analysis

All statistical analyses were performed using Empower Stats (https://www.empowerstats.net/cn/) and SPSS version 27.0. Categorical variables were presented as percentages and frequencies, while baseline characteristics between participants with and without WPV were compared using chi‐square tests. Participants were stratified into four groups based on their total WPV scale scores: none (0), low (1–5), medium (6–10), and high (11–15). Depression, anxiety, and stress were treated as continuous variables, while burnout and turnover intention were treated as dichotomous variables.

To investigate the relationships between WPV and various predictors, we employed hierarchical modeling approaches tailored to each outcome variable type. For the four‐level WPV categorical outcome, hierarchical linear regression models were fitted to examine associations with demographic variables, occupational characteristics, depression, anxiety, stress, burnout, and turnover intention. For the dichotomous outcomes of burnout and turnover intention, hierarchical multivariable logistic regression models were utilized. The four levels of WPV were defined as follows: level 1 corresponds to no violence, level 2 represents low violence (1–5), level 3 represents medium violence (6–10), and level 4 represents high violence (11–15).

The hierarchical modeling strategy involved three progressive adjustment levels. Model 1 included unadjusted analyses, Model 2 adjusted for demographic variables including sex, age, education level, and marital status, and Model 3 incorporated additional work‐related characteristics such as professional title, nightshift work, type of work, years of experience, and weekly working hours alongside the demographic adjustments from Model 2. Results from the adjusted logistic regression models are presented as adjusted odds ratios with 95% confidence intervals, with statistical significance set at *p* < 0.05. Missing data for covariates, including Age, BMI, Years of experience, and Weekly working hours, were addressed using expectation maximization imputation.

## 3. Results

### 3.1. Demographics

Table 1 shows the result of differences in WPV among these sociodemographic and work‐related characteristics. Of the 116,345 nurses, 26.6% (30,987) reported WPV in the previous year; 23.4% of nurses were exposed to low level of WPV, 3% were exposed to medium level of WPV, and 0.2% were exposed to a high level of WPV. Nurses who experienced WPV were significantly different from those who were not exposed to WPV in all sociodemographics and work‐related variables (*p* < 0.05).

**Table 1:** Characteristics and comparison of the groups with and without workplace violence in the past year.

Variables		Total (*n* = 116,345, 100%)	Without WPV (*n* = 85,358, 73.4%)	With WPV (*n* = 30,987, 26.6%)	*p* value
Sex	Male	7653 (6.6%)	5783 (75.6%)	1870 (24.4%)	< 0.001
	Female	108,692 (93.4%)	79,575 (73.2%)	29,117 (26.8%)	

Age group (years)	< 30	42,696 (36.7%)	30,943 (72.5%)	11,753 (27.5%)	< 0.001
	30–40	55,697 (47.9%)	40,753 (73.2%)	14,944 (26.8%)	
	> 40	17,952 (15.4%)	13,662 (76.1%)	4290 (23.9%)	

Level of education	Below bachelor’s degree	9958 (8.6%)	7718 (77.5%)	2240 (22.5%)	< 0.001
	Bachelor’s degree	101,380 (87.1%)	74,117 (73.1%)	27,263 (26.9%)	
	Master’s degree or above	5007 (4.3%)	3523 (70.4%)	1484 (29.6%)	

Marital status	Married or cohabitating	80,218 (68.9%)	59,676 (74.4%)	20,542 (17.6%)	< 0.001
	Separated, widowed, or divorced	3079 (2.6%)	2139 (69.5%)	940 (30.5%)	
	Never married	33,048 (28.4%)	23,543 (71.2%)	9505 (28.8%)	

Professional title	Junior	60,000 (51.6%)	44,291 (73.8%)	15,709 (26.2%)	< 0.001
	Intermediate	50,587 (43.5%)	36,805 (72.8%)	13,782 (27.2%)	
	Senior	5758 (4.9%)	4262 (74%)	1496 (26%)	

Nightshift	No	28,207 (24.2%)	21,933 (77.8%)	6274 (22.2%)	< 0.001
	Yes	88,138 (75.8%)	63,425 (72%)	24,713 (28%)	

Type of work	Direct patient care	109,050 (93.7%)	79,853 (73.2%)	29,197 (26.8%)	< 0.001
	Nondirect patient care	7295 (6.3%)	5505 (75.5%)	1790 (24.5%)	

Work Experience (years)	< 10	63,022 (54.2%)	45,749 (72.6%)	17,273 (27.4%)	< 0.001
	10–20	39,917 (34.3%)	29,397 (73.6%)	10,520 (26.4%)	
	> 20	13,406 (11.5%)	10,212 (76.2%)	3194 (23.8%)	

Weekly working hours	< 40	80,489 (69.2%)	60,249 (74.9%)	20,240 (25.1%)	< 0.001
	40–50	29,328 (25.2%)	20,344 (69.4%)	8984 (30.6%)	
	> 50	6528 (5.6%)	4765 (73%)	1763 (27%)	

### 3.2. Hierarchical Linear Regression Analysis of WPV Levels and Their Effects on Mental Health

Table 2 presents the findings of the multivariable linear regression analysis on the association between WPV levels and anxiety, depression, and stress. Our research indicated that WPV was positively associated with mental health issues. In the crude model l, a high level of WPV in the past year exhibited higher mental health scores (PHQ‐9 score: *β* = 6.11, 95% CI 5.57 ∼ 6.65; GAD‐7 score: *β* = 4.96, 95% CI: 4.49 ∼ 5.42; PSS‐4 score: *β* = 2.06, 95% CI: 1.73 ∼ 2.39) compared to those unexposed to WPV. Moreover, as WPV levels increased, mental health scores gradually increased (*p* for trend < 0.001). The associations between low, medium, and high levels of WPV and mental health were significant in all models, even after hierarchically adjusting the demographic variables in Model 2 and workplace‐related variables in Model 3. In Model 3, compared to participants unexposed to WPV, participants in the high WPV group had higher PHQ‐9 scores (*β* = 6.05, 95% CI: 5.51 ∼6.59), GAD‐7 scores (*β* = 4.95, 95% CI: 4.49 ∼ 5.41), and PSS‐4 scores (*β = *2.00, 95% CI: 1.68 ∼ 2.33). Participants in the low and medium WPV groups also exhibited significantly higher PHQ‐9 scores (*p* for trend < 0.001), GAD‐7 scores (*p* for trend < 0.001), and PSS‐4 scores (*p* for trend < 0.001) than those who never experienced WPV.

**Table 2:** Association between WPV levels and depression, anxiety, and pressure scores.

Variables	WPV	Model 1		Model 2		Model 3	
		*β* (95% CI)	*p* value	*β* (95% CI)	*p* value	*β* (95% CI)	*p* value
Depressive symptoms	Never	Ref		Ref		Ref	
	Low	1.93 (1.88–1.99)	< 0.001	1.92 (1.86–1.98)	< 0.001	1.89 (1.83–1.95)	< 0.001
	Medium	3.96 (3.82–4.11)	< 0.001	3.94 (3.79–4.08)	< 0.001	3.89 (3.75–4.04)	< 0.001
	High	6.11 (5.57–6.65)	< 0.001	6.11 (5.57–6.65)	< 0.001	6.05 (5.51–6.59)	< 0.001
	*P* for trend		< 0.001		< 0.001		< 0.001

Anxiety symptoms	Never	Ref		Ref		Ref	
	Low	1.54 (1.49–1.59)	< 0.001	1.54 (1.49–1.59)	< 0.001	1.52 (1.47–1.57)	< 0.001
	Medium	3.07 (2.95–3.19)	< 0.001	3.07 (2.94–3.19)	< 0.001	3.03 (2.91–3.16)	< 0.001
	High	4.96 (4.49–5.42)	< 0.001	5.00 (4.53–5.46)	< 0.001	4.95 (4.49–5.41)	< 0.001
	*P* for trend		< 0.001		< 0.001		< 0.001

Perceived stress	Never	Ref		Ref		Ref	
	Low	0.93 (0.89–0.96)	< 0.001	0.92 (0.88–0.95)	< 0.001	0.91 (0.88–0.95)	< 0.001
	Medium	1.68 (1.59–1.77)	< 0.001	1.66 (1.57–1.75)	< 0.001	1.66 (1.57–1.74)	< 0.001
	High	2.06 (1.73–2.39)	< 0.001	2.02 (1.70–2.36)	< 0.001	2.00 (1.68–2.33)	< 0.001
	P for trend		< 0.001		< 0.001		< 0.001

*Note:* Model 1: Unadjusted. Model 2: Adjusted for sex, age, education level, and marital status. Model 3: Adjusted for all variables in Model 2, additionally adjusted for professional title, night shift, type of work, years of experience, and weekly working hours. *β*, unstandardized regression coefficients.

Abbreviations: 95% CI, 95% confidence interval; WPV, workplace violence.

### 3.3. Hierarchical Multivariable Logistic Regression Analysis of WPV Levels and Their Effects on Burnout, Turnover Intention

All the results of multiple models with different variables suggested significant associations between three levels of WPV and burnout, and turnover intention. After multivariate analysis (Model 3), adjusted ORs of burnout among participants who experienced low, medium, and high levels of WPV were 1.93 (95% CI, 1.87–1.98), 3.56 (95% CI, 3.31–3.83), and 5.16 (95% CI, 3.84–6.93), respectively, compared with those who did not experience WPV in the past year. For turnover intention, after multivariate analysis (Model 3), the adjusted ORs among participants who experienced low, medium, and high levels of WPV were 2.18 (95% CI, 2.11–2.26), 3.38 (95% CI, 3.05–3.74), and 5.11 (95% CI, 3.25–8.05), respectively. Details are shown in Table 3.

**Table 3:** Association between WPV levels and burnout, turnover intention.

Variables	WPV	Model 1		Model 2		Model 3	
		OR (95% CI)	*p*	OR (95% CI)	*p*	OR (95% CI)	*p*
Burnout	Never	Ref		Ref		Ref	
	Low	1.96 (1.90–2.01)	< 0.001	1.95 (1.90–2.00)	< 0.001	1.93 (1.87–1.98)	< 0.001
	Medium	3.62 (3.37–3.90)	< 0.001	3.59 (3.34–3.86)	< 0.001	3.56 (3.31–3.83)	< 0.001
	High	5.22 (3.89–7.00)	< 0.001	5.21 (3.89–6.99)	< 0.001	5.16 (3.84–6.93)	< 0.001
	*P* for trend		< 0.001		< 0.001		< 0.001

Turnover intention	Never	Ref		Ref		Ref	
	Low	2.23 (1.16–2.31)	< 0.001	2.21 (2.14–2.29)	< 0.001	2.18 (2.11–2.26)	< 0.001
	Medium	3.25 (2.94–3.60)	< 0.001	3.34 (3.02–3.70)	< 0.001	3.38 (3.05–3.74)	< 0.001
	High	4.98 (3.18–7.79)	< 0.001	5.14 (3.28–8.08)	< 0.001	5.11 (3.25–8.05)	< 0.001
	*P* for trend		< 0.001		< 0.001		< 0.001

*Note:* Model 1: Unadjusted. Model 2: Adjusted for sex, age, education level, and marital status. Model 3: Adjusted for all variables in Model 2, additionally adjusted for professional title, night shift, type of work, years of experience, and weekly working hours.

Abbreviations: 95% CI, 95% confidence interval; OR, odds ratio; WPV, workplace violence.

## 4. Discussion

The results showed that 26.6% of nurses experienced at least one form of WPV during the past 12 months, a rate substantially lower than previous studies conducted in China, which reported prevalence rates ranging from 41.75% to 55% [37–39]. This finding is also notably lower than the 58% prevalence reported in a meta‐analysis of 36,566 nurses across the Western Pacific and Southeast Asian regions [40] and the 43% rate found in a meta‐analysis of 27,030 nurses from the United States [41]. Several methodological and contextual factors may explain these variations in reported prevalence rates. Many previous studies examining WPV rates relied on convenience sampling methods, which tend to yield higher reported instances of violence, particularly when conducted in psychiatric settings where nurses face heightened vulnerability to violent encounters. In contrast, all participants in this study were recruited exclusively from tertiary hospitals, which typically maintain more robust safety protocols and have greater resources available for violence prevention and response. Additionally, recent policy changes in China, including stricter government penalties for WPV against HCWs, may have contributed to creating safer work environments and deterring violent behavior [42].

The timing of data collection also represents a crucial contextual factor that may have influenced these findings. Data were gathered between October and December 2023, a period still reflecting the unique circumstances and aftermath of the COVID‐19 pandemic. During this time, nurses experienced increased public recognition and social support for their contributions to pandemic response efforts, which may have led to a reduction in instances of WPV. Furthermore, while the pandemic initially led to increased patient volumes in emergency and intensive care settings, it simultaneously resulted in reduced patient visits for nonurgent care due to lockdown measures and modified hospital policies. These pandemic‐related changes in healthcare delivery patterns and public attitudes toward HCWs may have contributed to the lower incidence of WPV observed in this study compared to prepandemic reports. Additionally, it is important to note that the total sample (*N* = 116,345) provides robust power, and the high‐level WPV subgroup (*n* = 243, 0.2%) limits statistical precision for extreme exposure estimates. We addressed this through multilevel modeling (adjusting for nesting/confounders) and effect size reporting. Consequently, high‐WPV associations require cautious interpretation. Future studies must prioritize larger high‐exposure cohorts with longitudinal designs to validate these effects. Our research emphasizes the critical need for specific interventions to reduce WPV and its adverse impacts on the mental health of nurses in China.

Although the prevalence of WPV in this study was lower than reported in other research, its relationship with mental health outcomes remains profoundly significant. Our findings demonstrated that nurses with higher levels of exposure to WPV were more likely to develop negative health outcomes, including depression symptoms, anxiety symptoms, stress, burnout, and turnover intention. By categorizing WPV into four distinct levels, none, low, medium, and high, this study provided a quantitative framework for examining the relationship between WPV exposure and nurses’ mental health outcomes. While direct coefficient comparisons with previous research were not feasible due to methodological differences, the associations identified in this study align consistently with existing literature demonstrating that WPV serves as a significant risk factor for depression symptoms, anxiety symptoms, stress, and burnout [43–45]. The Chinese healthcare context presents unique challenges that may intensify these relationships, as nurses face increasing clinical workloads driven by growing patient numbers and demanding health policy requirements. Their demanding schedules and expanding responsibilities leave limited time for processing and releasing negative emotions and stress arising from WPV experiences, ultimately contributing to the development of serious psychological problems [46, 47].

Theoretical frameworks provide valuable insight into these observed relationships. The Challenge‐Hindrance Stress Model explains how individuals experience both physical and psychological stress responses when confronted with threatening events or attacks, while the Conservation of Resources Theory clarifies that human resources are finite [48]. WPV poses a direct threat to personal safety and triggers defensive mechanisms that can result in trauma, resistance, and an increase in negative emotions. The psychological and emotional reserves required to cope with violent events may become gradually depleted, eventually leading to job burnout and even intentions to leave the profession [49]. This progression aligns with prior research findings that identified WPV as a positive predictor of turnover intention [30], with our study revealing that individuals experiencing high levels of WPV are five times more likely to leave their positions. Affective events theory provides additional theoretical grounding for these findings by explaining how work environment characteristics trigger either positive or negative workplace events, which subsequently cause emotional reactions that influence individual attitudes and behaviors [50]. Specifically, exposure to WPV generates heightened negative emotions among nurses, which may substantially increase their intention to quit their positions. These findings underscore the critical importance of recognizing mental health risks and implementing comprehensive policies and strategies for all nurses as a significant retention approach across healthcare settings.

Our research emphasizes the critical need for developing and implementing specific interventions to reduce WPV and mitigate its adverse impacts on nurses’ mental health in China. Effective interventions should be implemented across multiple levels, national, hospital, and individual, to create comprehensive protection against WPV. At the national level, policymakers should enact stronger legislation and reinforce existing laws to safeguard nurses from aggressive behavior. Hospital administrators should implement practical safety measures such as installing surveillance cameras and ensuring adequate security presence, particularly in departments where nurses are more vulnerable to violence encounters [51, 52].

Healthcare institutions must establish accessible, efficient, and confidential WPV prevention programs and reporting systems that are essential for both preventing violence and managing the aftermath of incidents [53]. Nurse managers should be encouraged to cultivate a culture of open reporting and promote a just culture that supports incident reporting without fear of retribution. Following WPV incidents, hospitals should acknowledge nurses’ professional dedication and provide comprehensive institutional and managerial support. Supervisors should be empowered to make informed decisions regarding modifications to work locations, duties, and shifts to reduce the likelihood of subsequent violence episodes [53]. Providing supportive follow‐up care, including referrals to mental health professionals when necessary, is a crucial component of postincident support [54]. At the individual level, communication skills training can enhance patient–nurse relationships and potentially reduce the occurrence of WPV [12].

## 5. Strengths and Limitations

This study demonstrates several notable strengths that enhance the validity and reliability of our findings. The large sample size provided robust statistical power and was representative of the Chinese nursing population with respect to key demographic characteristics, strengthening the external validity of our results. Another notable strength is the introduction of a graded exposure framework, which categorizes WPV into four severity levels (none, low, medium, and high). This stratification allows for the quantification of dose–response relationships between WPV intensity and mental health outcomes such as depression, anxiety, stress, and turnover intention. High‐level WPV increased burnout risk 5.16‐fold and turnover intention 5.11‐fold versus unexposed nurses, establishing unprecedented thresholds for intervention targeting. By offering a more detailed analysis of the impact of WPV, the study advances the field beyond simplistic categorizations used in previous research. Methodologically, the study employs hierarchical multilevel regression models, which account for confounding variables such as sociodemographic and occupational factors. This allows for a more precise isolation of WPV severity as an independent predictor of mental health, addressing limitations found in previous single‐center studies.

However, several important limitations must be acknowledged when interpreting these findings. The cross‐sectional design of the study fundamentally precludes the establishment of causal relationships, limiting our ability to infer causation from the observed associations between WPV and mental health outcomes. Future longitudinal prospective studies are essential to examine the long‐term effects of WPV on nurses’ psychological health and to establish temporal relationships between exposure and outcomes. Despite the substantial sample size, the reliance on online survey methodology may have introduced bias through self‐reporting and recall limitations, as participants may consciously or unconsciously conceal sensitive information or experience difficulty accurately recalling specific incidents of WPV.

Furthermore, the study’s focus exclusively on nurses employed in tertiary hospitals may not adequately capture the full spectrum of WPV experiences encountered by nurses across different healthcare settings. Tertiary hospitals typically have more robust safety protocols, greater resources, and different patient populations compared to primary care facilities, community hospitals, or specialized settings, which may result in different patterns and frequencies of WPV exposure. This sampling limitation restricts the generalizability of our findings to the broader nursing workforce. It underscores the need for future research to encompass diverse healthcare settings, thereby developing a more comprehensive understanding of WPV experiences across the nursing profession. To address this, future research should prioritize stratified sampling across diverse healthcare settings, including community health centers and primary care clinics. Additionally, the Cronbach’s α of 0.657 for the Perceived Stress Scale‐4 (PSS‐4) suggests moderate reliability, which is considered acceptable for ultrashort scales in exploratory research. However, this may affect the precision of stress measurement. Future studies should consider using more reliable scales or further validating the PSS‐4 in diverse nursing populations.

## 6. Conclusion

In summary, repeated exposure to WPV events has a cumulative effect on adverse mental health symptoms and turnover intention. In nursing management, specific strategies are crucial for addressing the causes of WPV and reducing its negative impacts. Developing effective intervention protocols at the individual, institutional, and national levels is crucial for effectively managing WPV. To prevent negative effects on their mental health, it is essential to observe nurses’ emotional reactions and support them. Future longitudinal research using nationally representative samples is necessary to substantiate further the relationship between WPV, nurses’ mental health, and turnover intention.

## Ethics Statement

This study was conducted in accordance with the principles of the Declaration of Helsinki and received approval from the Ethics Committee of the Second Xiangya Hospital of Central South University (Approval No: LYF20230048).

## Disclosure

All authors approved the final version of the manuscript for publication.

## Conflicts of Interest

The authors declare no conflicts of interest.

## Author Contributions

All authors participated in reviewing subsequent drafts. Jiaqing He, Jiaxin Yang, Qiang Yu, and Yusheng Tian assisted in the collection of data from each of the participating centers; Jiaqing He, Jiaxin Yang, and Jianghao Yuan led the development of the study design; Qiang Yu analyzed and interpreted the data; Jiaqing He drafted the manuscript; Elihuruma E. Stephano and Wenjia Zhang assisted in refining writing details; and Yamin Li and Yusheng Tian were responsible for the supervision, validation, and critical revision of the manuscript.

## Funding

This study was supported by STI2030‐Major Projects (2021ZD0200700), Major Scientific and Technological Projects in Hunan Province (2020SK2085), the Chinese Nursing Association (ZHKY202306), Hunan Provincial People′s Hospital (KCTL202502), and Special Funding for Leading Disciplines—Clinical Nursing (XK202003L).

## Supporting Information

Supporting Information includes the STROBE statement, which provides a checklist of the reporting guidelines followed in this observational study. Additionally, a conflict of interest statement is provided.

## Supporting information


**Supporting Information** Additional supporting information can be found online in the Supporting Information section.

## Data Availability

The data supporting the findings of this study are available upon request from the corresponding author. The data are not publicly available due to privacy or ethical restrictions.
